# A20 deficiency leads to angiogenesis of pulmonary artery endothelial cells through stronger NF‐κB activation under hypoxia

**DOI:** 10.1111/jcmm.12816

**Published:** 2016-03-17

**Authors:** Jing Li, Linlin Zhang, Yueming Zhang, Ying Liu, Hongyue Zhang, Liuping Wei, Tingting Shen, Chun Jiang, Daling Zhu

**Affiliations:** ^1^ Department of Biopharmaceutical SciencesHarbin Medical UniversityDaqingHeilongjiangChina; ^2^ Department of Biopharmaceutical Key Laboratory of Heilongjiang ProvinceHarbin Medical UniversityHarbinHeilongjiangChina; ^3^ Department of BiologyGeorgia State UniversityAtlantaGAUSA

**Keywords:** A20, hypoxia, angiogenesis, pulmonary artery endothelial cells, nuclear factor‐kappa B

## Abstract

A20 is a zinc finger protein associated with hypoxia. As chronic hypoxia is responsible for intimal hyperplasia and disordered angiogenesis of pulmonary artery, which are histological hallmarks of pulmonary artery hypertension, we intended to explore the role of A20 in angiogenesis of pulmonary artery endothelial cells (ECs). Here, we found a transient elevation of A20 expression in the lung tissues from hypoxic rats compared with normoxic controls. This rapid enhancement was mainly detected in the endothelium, and similar results were reproduced *in vitro*. During early hypoxia, genetic inhibition of A20 increased proliferation in pulmonary artery ECs, linking to advanced cell cycle progression as well as microtubule polymerization, and aggravated angiogenic effects including tube formation, cell migration and adhesion molecules expression. In addition, a negative feedback loop between nuclear factor‐kappa B and A20 was confirmed. Our findings provide evidence for an adaptive role of A20 against pulmonary artery ECs angiogenesis *via* nuclear factor‐kappa B activation.

## Introduction

Pulmonary artery hypertension (PAH) is a progressive pulmonary vascular disease, which has been characterized by vasoconstriction, vascular remodelling, increased vascular resistance, and leads to right heart failure and death 1, 2, 3. To date there is no effective therapeutical modality for curing the disease, and the mortality rate of PAH patients is fairly high. Therefore, elucidating the molecular and cellular basis underlying the pathogenesis of PAH, and exploring new therapeutic strategies are quite necessary 4.

It is well known that PAH is originated from aberrant vasculature consisting of endothelial cells (ECs), smooth muscle cells (SMCs) and fibroblasts 1. Excessive proliferation, migration, adhesion and tube formation of ECs have been demonstrated to promote new vessel growth and angiogenesis, conduce to intimal hyperplasia and vascular abnormalities 5, 6, 7, 8, 9. Despite hyperproliferative ECs and disordered angiogenesis are quite crucial for vascular remodelling in chronic lung diseases such as PAH 8, 9, the mechanisms regulating hypoxia‐induced angiogenesis are yet not completely been elucidated.

One potential regulatory factor during the angiogenic pathogenesis is presumed to be A20. In human umbilical vein ECs, A20 is first defined as a novel type of zinc finger protein regulated by tumour necrosis factor (TNF)‐α 10, 11. Subsequent studies indicate that A20 is widely expressed in multiple cell types, including aortic ECs, SMCs, breast cancer cells, B cells and dendritic cells 12, 13, 14, 15, 16, 17, 18, 19. However, the precise role for A20 in proliferation and apoptosis is controversial, depending on specific stimulus and cell types. For instance, with the treatment of 17β‐estradiol, over‐expressed A20 exerts anti‐apoptosis activity in breast cancer cells 8. In contrast, dendritic cells lacking A20 show a proliferative and apoptosis‐resistant phenotype 13. Identification of the clear role for A20 in different environments is crucial to improve our understanding.

Recent reports reveal that A20 is hypoxia‐inducible in glioblastoma cell‐lines and macrophages 20, 21. In addition, nuclear factor‐kappa B (NF‐κB) as a capital transcription factor sensitive to hypoxia in pulmonary artery endothelial cells (PAECs), is required for A20 transcriptional activation 22, 23. These findings raise the possibility of hypoxia induces A20 through NF‐κB in PAECs.

In this study, the functional role of A20 in hypoxic PAECs was investigated. Our results showed a rapid enhancement of A20 by hypoxia both *in vivo* and *in vitro*. Under hypoxia exposure, A20 down‐regulation led to proliferation and angiogenesis of PAECs *via* a stronger activation of NF‐κB.

## Materials and methods

For detailed Materials and methods, please see the online Data S1.

### Animal model

Adult male Wistar rats at a mean weight of 200 g were obtained from the Experimental Animal Center of Harbin Medical University, China. Animals were divided into groups randomly, and kept in normoxic or hypoxic environment as we reported previously. The oxygen concentrations used for normoxia group were 21%, and for hypoxia was 12% 24. At the end of exposure, animals were killed after anaesthesia, and lung tissues were obtained for further studies. Permission from the Animal Care and Use Committee of Harbin Medical University was received, and all animal procedures were performed according to the guidelines of the Animal Care and Use Committee.

### Cell culture

Calf lung tissues obtained from an authorized Slaughterhouse were approved by the Ethical Committee of Laboratory Animals at Harbin Medical University. Permission from the Institutional Animal Care and Use Committee was received, and all animal procedures were performed according to the guidelines of the Institutional Animal Care and Use Committee. The primary culture of PAECs was performed by gentle scraping along the intimal surface of vessels with a surgical blade, which we have described before 25.The purity of PAECs was verified using antibody specific to PECAM by immunocytochemistry. Cells were cultured with DMEM containing 20% FBS serum, and passage 2–3 were used for further experiments. As we reported previously, cells were incubated in a humidified incubator (Thermo Fisher, Runcorn, Cheshire, UK) with 5% CO_2_ at 37°C for normoxic conditions, and a Tri‐Gas incubator (Heal Force, Shanghai, China) providing 3% O_2_/5% CO_2_/92% N_2_ was applied for hypoxic cultivation 26, 27.

### Statistical analysis

All data are expressed as mean ± S.E.M. Statistical significance was determined with one‐way anova, followed by Dunnett's test or Student's *t*‐test. Differences were considered to be significant at *P* < 0.05.

## Results

### A20 is rapidly induced in pulmonary artery endothelium by hypoxia both *in vivo* and *in vitro*

To examine the expression and distribution of A20 in lung vasculature, hypoxic rat model was constructed. Haematoxylin‐eosin stainings and α‐SMA protein expression were determined to assess the histological changes of blood vessels. From the 3rd day to the 5th day of hypoxia treatment, there was no difference compared with normoxic group (hypoxia exposure for 0 day; Fig. S1A and B). At the 9th day of hypoxia, we noted enhanced α‐SMA expression and thickened vessels (Fig. S1A and B). It was previously reported that exposure to hypoxia for 9 days could conduce to vascular abnormalities, increase right ventricular mass, and has been used as a model of PAH in several studies 25, 27, 28, 29, 30, 31.

**Figure 1:**
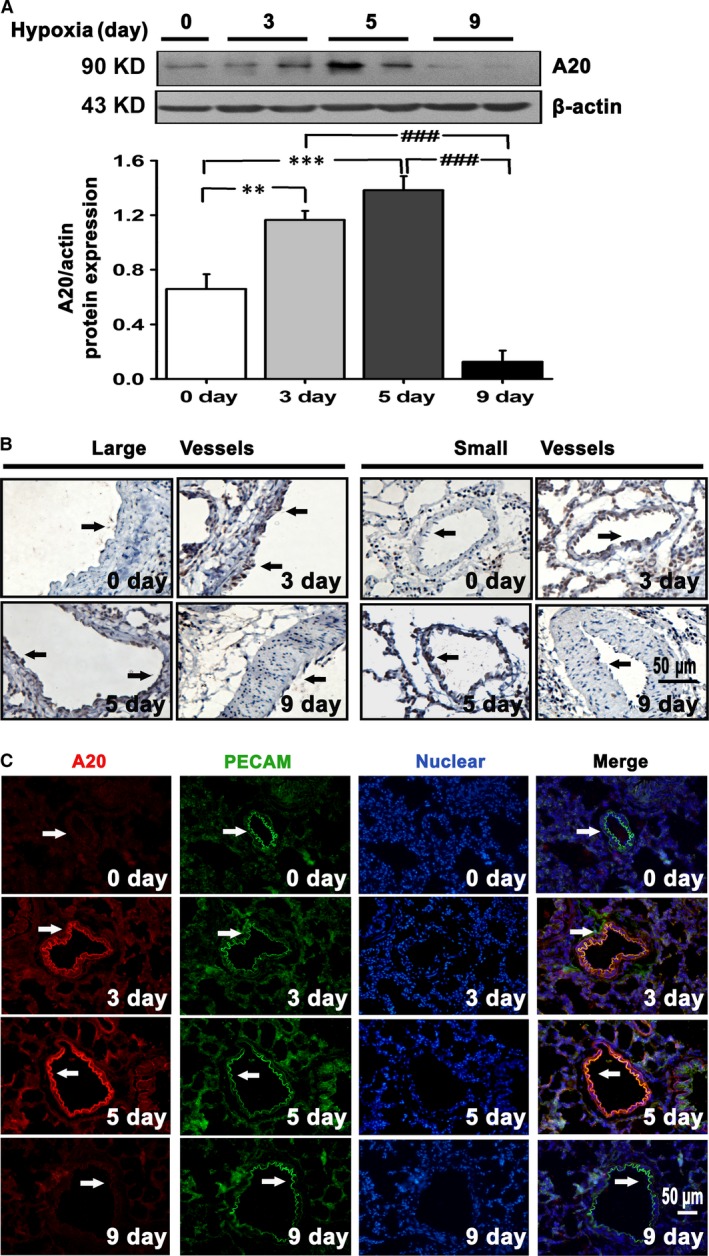
Expression of A20 is enhanced in pulmonary artery endothelium of hypoxic rats. (**A**) Quantification of A20 protein levels in the lung tissues from rats exposed to hypoxia for different days (0, 3, 5, 9 days; *n* = 4, **P < 0.01, ***P < 0.001, ###*P* < 0.001). All the values are represented as mean ± S.E.M. (**B**) Immunohistochemical staining of A20 in large and small pulmonary arteries. (**C**) Lung sections were stained for A20 with Cy3‐conjugated secondary antibodies (red colour), PECAM with FITC‐conjugated secondary antibodies (green colour), and nucleus with DAPI (blue colour). Merged images show A20 colocalizes to PECAM. Arrows indicate positive stainings, scale bars are 50 μm. Images shown are representative of at least three independent experiments.

The protein levels of A20 in lung tissues from hypoxic rats were much higher from the 3rd day to the 5th day. Following this increase, the expression of A20 declined to a lower level in both large and small resistance vessels at the 9th day, accompanied by a thickening of blood vessels (Fig. 1A and B). Given the vascular changes during hypoxia, these findings suggest a possible correlation between A20 expression and disordered vasculature in a hypoxia‐dependent manner. The findings in Figure 1C that the majority of elevated A20 colocalized with PECAM (a marker of ECs), suggested that A20 distributed mainly in endothelium. Therefore, the *in vitro* studies were performed with cultured PAECs.

**Figure 2:**
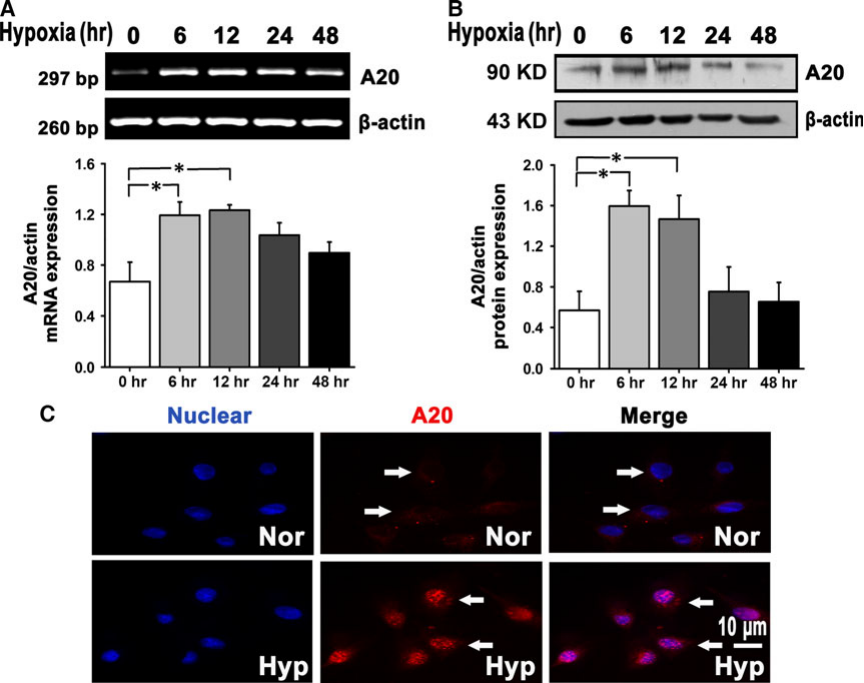
A20 mRNA and protein expression is promoted in hypoxic PAECs. (**A** and **B**) PCR and western blot analysis of A20 expression at different time‐points under hypoxia (0, 6, 12, 24, 48 hrs; *n* = 4, *P < 0.05). All of the values are represented as mean ± S.E.M. (**C**) PAECs were exposed to normoxia or hypoxia for 12 hrs and A20 location was examined by immunocytochemistry. Red colour represents A20 stained with Cy3, blue colour denotes nucleus stained with DAPI. Arrows indicate positive stainings, scale bar represents 10 μm. Images shown are representative of at least three independent experiments. hr: hour; Nor: normoxia; Hyp: hypoxia.

A20 mRNA and protein expression under hypoxia was further investigated in Figure 2A and B. The results showed an increase of A20, which peaked at 6–12 hrs and fell at 24–48 hrs. In addition, it was observed by immunofluorescence assay that elevated A20 by hypoxia was in cellular nucleus (Fig. 2C). These data show that hypoxia induces a rapid increase of A20 in pulmonary artery endothelium, and promotes its accumulation in nucleus.

### A20 repression contributes to proliferation of PAECs under hypoxia

To explore the potential function of A20 under hypoxia, specific siRNA and expression vector were transfected into PAECs. The efficiency was verified by western blot (Figs S2A and S3A).

**Figure 3:**
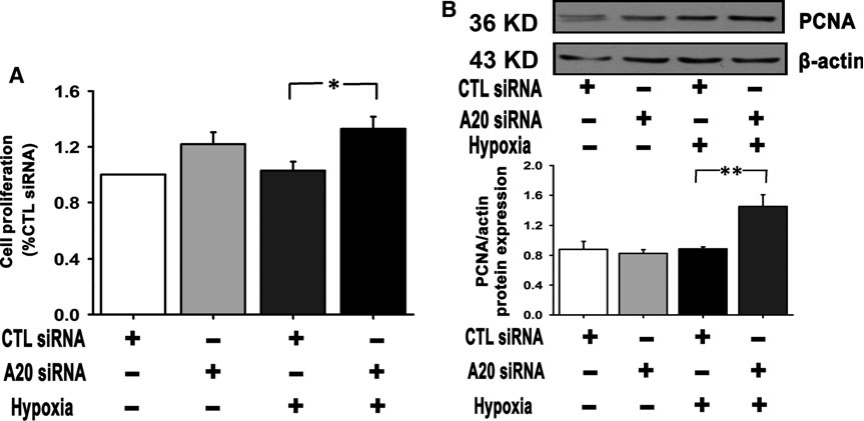
A20 modulates proliferation of PAECs during hypoxia (for 12 hrs). (**A**) PAECs transfected with CTRL siRNA or A20 siRNA were exposed to normoxia or hypoxia. Proliferation of PAECs was assessed by BrdU incorporation assay (*n* = 8, *P < 0.05). Data were expressed as relative values normalized to the CTL siRNA group. (**B**) PCNA expression was determined by western blot analysis (*n* = 4, **P < 0.01). All the values are represented as mean ± S.E.M.

We measured the cell proliferation by BrdU incorporation assay and found that during the prophase of hypoxia (12 hrs), when A20 expression was increased, there was no significant difference between normoxic and hypoxic groups. A20 siRNA led to increased cell proliferation compared to CTRL siRNA under hypoxia (Fig. 3A). Consistently, under sustained hypoxia (24 hrs), A20 down‐modulation induced an increase in cell viability, while A20 overexpression attenuated the level of cell viability (Figs S2B and S3B). Similarly, the cells exposed to transient hypoxia showed elevated proliferating cell nuclear antigen (PCNA) expression after A20 silencing (Fig. 3B). Taken together, we conclude that decreased A20 in hypoxic PAECs results in exuberant cell growth, and that high A20 expression levels help maintaining the cells in a normal state.

### Pivotal role of A20 in cell cycle progression and microtubule dynamic stability

**Figure 4:**
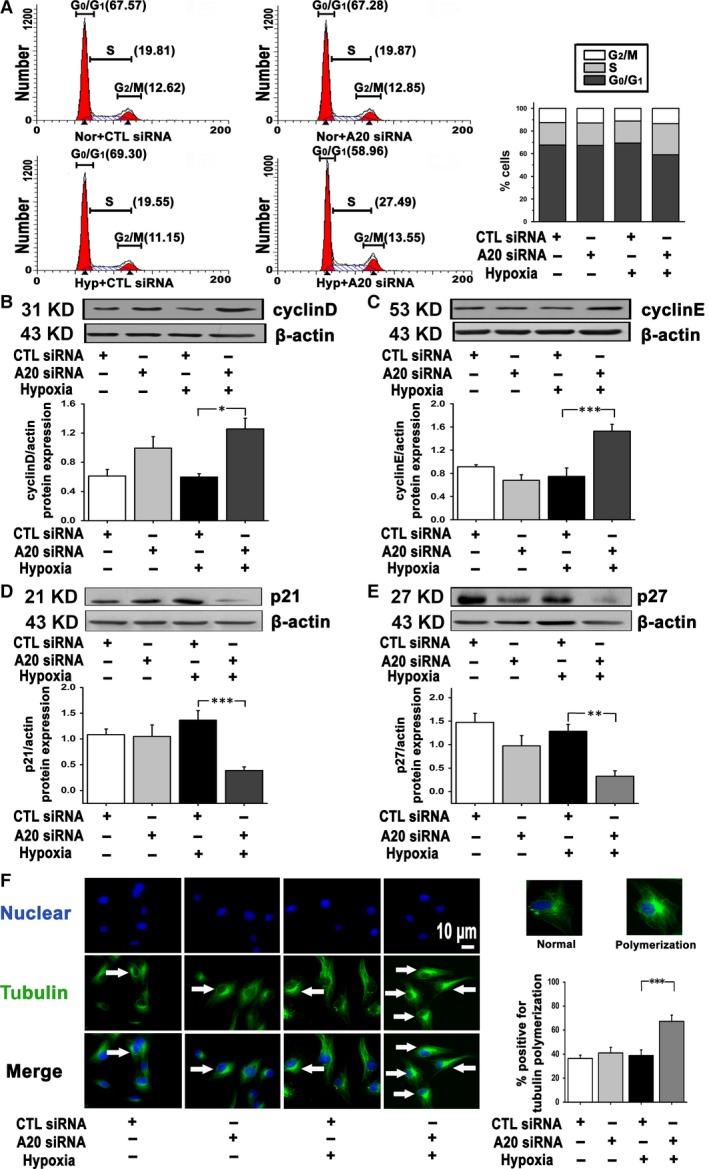
Suppressed A20 advances cell cycle progress, increases cell cycle‐related proteins, and promotes microtubule formation in PAECs subjected to hypoxia (12 hrs). (**A**) Upon the transfection of CTRL siRNA or A20 siRNA, cells were exposed to normoxia or hypoxia. The percentage of cells in G_0_/G_1_, S and G_2_/M phase of cell cycle was quantified by flow cytometer analysis (*n* = 2). (**B** and **C**) Western blot analysis of cyclin D and cyclin E expression (*n* = 3 and *n* = 5, *P < 0.05, ***P < 0.001). (**D** and **E**) Protein levels of p21 and p27 were analysed (*n* = 9 and *n* = 8, ***P < 0.001, **P < 0.01). (**F**) Cells were stained with anti‐α‐tubulin for microtubules (green colour), and DAPI for nucleus (blue colour). A quantification of the percentage for tubulin polymerization was shown (*n* = 13, ***P < 0.001). Arrows indicate microtubule polymerized around the nucleus, scale bar represents 10 μm. All the values are represented as mean ± S.E.M.

The effect of A20 on cell cycle progression was evaluated. We noted that upon A20 siRNA transfection, the percentage of S phase cells under hypoxia was increased from 19.55% to 27.49%, accompanied by a reduction in cells in the G_0_/G_1_phase from 69.3% to 58.96%, compared with cells treated with control siRNA (Fig. 4A). Cell cycle‐related proteins cyclin D and cyclin E were increased, while cyclin‐dependent kinase inhibitors p21 and p27 were decreased when A20 was interfered under hypoxic conditions (Fig. 4B–E). In addition, microtubule formation was assessed by tubulin staining. Under hypoxia cells lacking A20 overall displayed an increase amount of polymerized microtubules around the nucleus (Fig. 4F). These results confirm an important role of A20 in cell cycle progression and microtubule formation during hypoxia.

### A20 deficiency results in enhanced migration, tube formation and adhesion molecule expression

**Figure 5:**
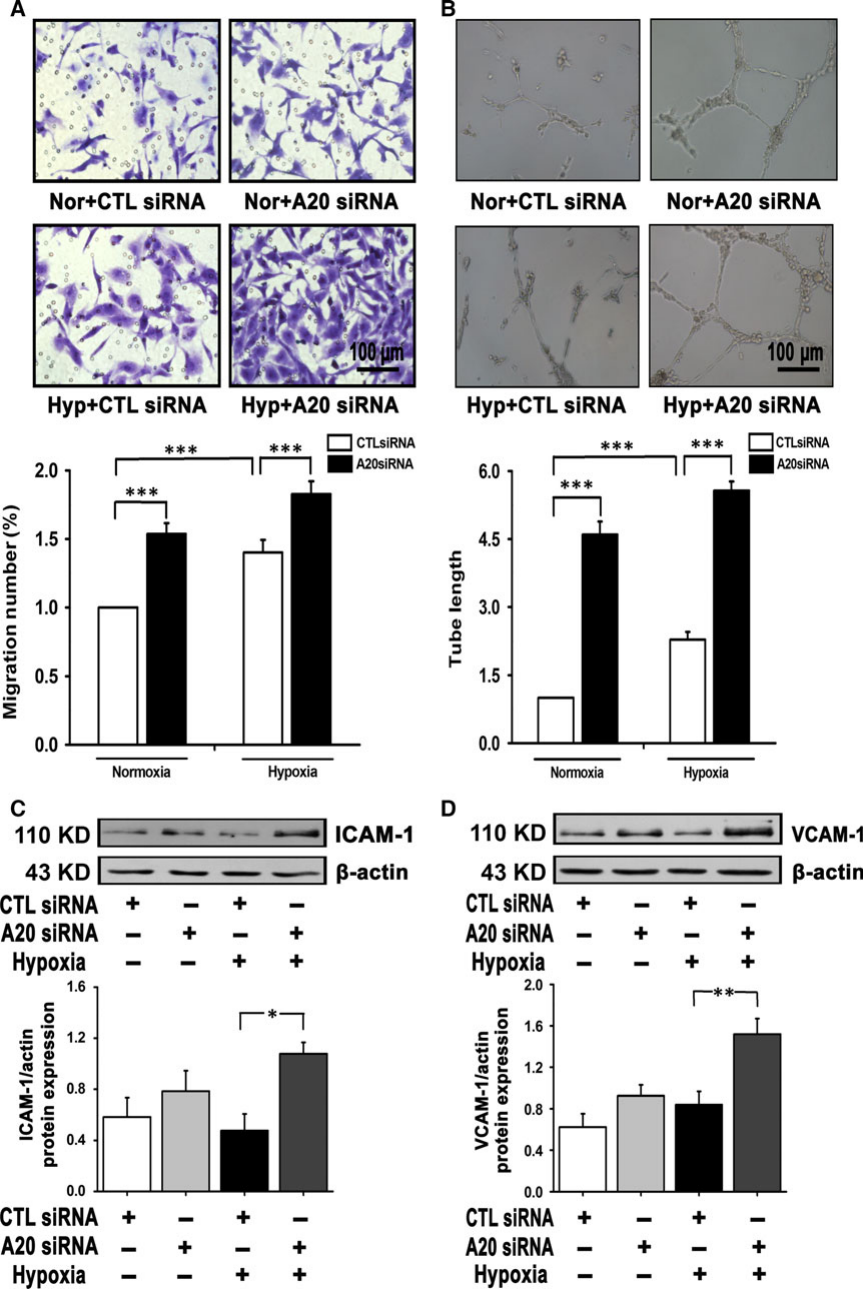
Migration, tube formation and expression of adhesion molecules in hypoxic PAECs are elevated after A20 suppresssion. (**A**) Boyden chamber migration assays was performed and the number of migratory cells under normoxia and hypoxia (12 hrs) was assessed by crystal violet staining (*n* = 13, ***P < 0.001). (**B**) Tube formation of PAECs was examined and quantified at 6–12 hrs of hypoxia (*n* = 8, ***P < 0.001). Relative values are normalized to control group, scale bars are 100 μm. (**C** and **D**) ICAM‐1 and VCAM‐1 protein levels were affected by siRNA targeting A20 (*n* = 4 and *n* = 7, *P < 0.05, **P < 0.01). Hypoxia time‐points was 12 hrs. All of the values are represented as mean ± S.E.M. Nor: normoxia; Hyp: hypoxia.

To investigate the effect of A20 in angiogenic events, we examined cell migration, tube formation and the expression of adhesion molecules. Notably, A20 siRNA induced higher number of migrated cells in compared to CTRL, suggesting a greater migratory capability by A20 inhibition (Fig. 5A). In Figure 5B, PAECs cultured on Matrigel formed capillary‐like tube structures. The tube length following A20 suppression was increased compared with control cells. In addition, enhanced expressions of cell adhesion molecules, including intercellular adhesion molecule‐1 (ICAM‐1) and vascular cell adhesion molecule‐1 (VCAM‐1) were observed when A20 was inhibited during hypoxia (Fig. 5C and D). Thus, it is elucidated that PAECs with attenuated A20 are more susceptible to form tube structures, to migrate and express adhesion molecules.

### A feedback loop between NF‐κB and A20 in hypoxic PAECs

It has been previously suggested that in Jurkat T cells, the up‐regulation of A20 transcript is mediated by NF‐κB activity 23. There are also evidence that A20 in aortic ECs blocks NF‐κB and modulates cell activation 32. Therefore, it was quite necessary to reveal the complicated relationship between A20 and NF‐κB in hypoxic PAECs.

**Figure 6:**
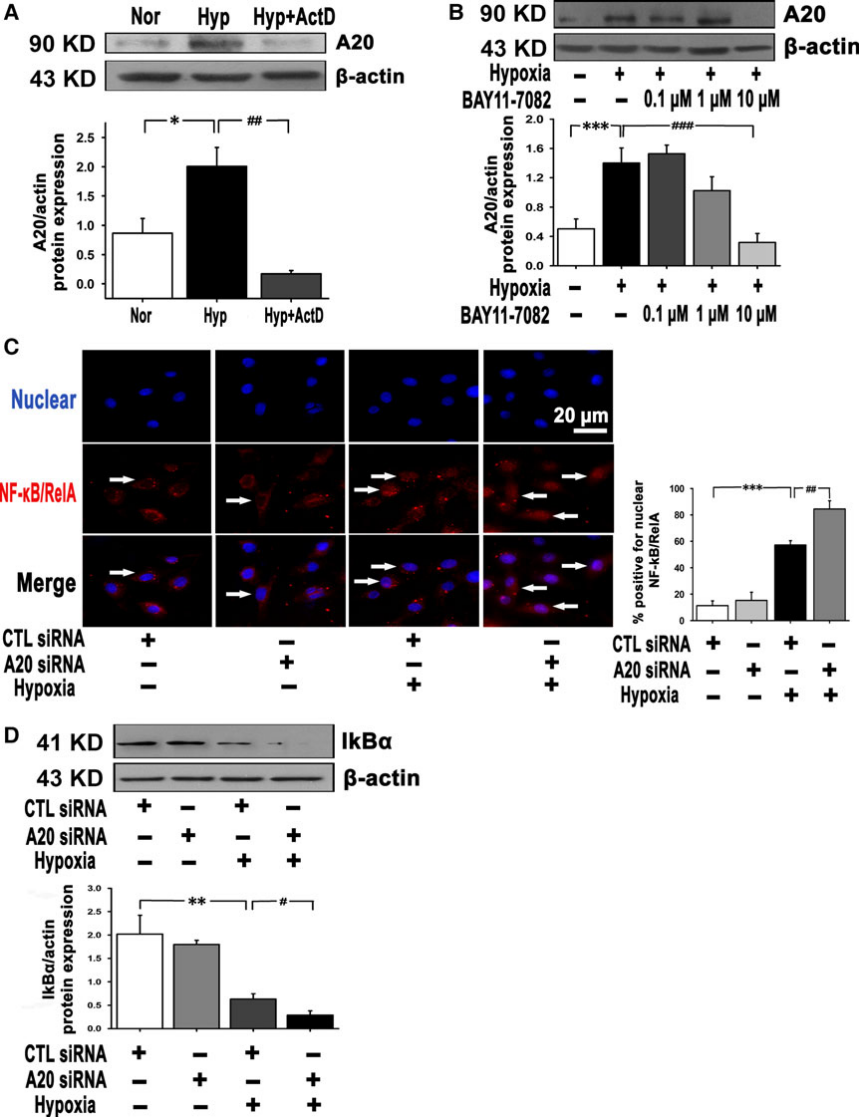
There is a reciprocal regulation between A20 and NF‐κB in PAECs under hypoxia. (**A**) Cells were pre‐incubated with actinomycinD (5 μmol/l) 1 hr before hypoxia (12 hrs), and A20 protein level was measured (*n* = 3, *P < 0.05, ##*P* < 0.01). (**B**) A20 expression was analysed in the presence of 0.1–10 μmol/l BAY11‐7082 under hypoxia (12 hrs) (*n* = 6, ***P < 0.001, ###*P* < 0.001). (**C**) Immunocytochemistry assay of NF‐κB/RelA translocation during hypoxia exposure (3 hrs). Red colour denotes NF‐κB/RelA by Cy3, and blue colour denotes nucleus by DAPI. A quantitive graph for the number of NF‐kB/RelA positive nuclei was shown (*n* = 8, ***P < 0.001, ##*P* < 0.01). Arrows indicate p65 stainings, scale bar represents 20 μm. (**D**) IκB‐α expression following hypoxia (3 hrs) was detected with A20 impaired (*n* = 3, **P < 0.01, #*P* < 0.05). All the values are represented as mean ± S.E.M. Nor: normoxia; Hyp: hypoxia; ActD: actinomycin D.

The increased A20 expression under hypoxia was blocked upon cell treatment with transcriptional inhibitor ActD (Fig. 6A). This suggests that there is a transcription‐dependent mechanism, and the expression of A20 is possibly regulated by transcription factors. Following that, we found that BAY11‐7082, an NF‐κB inhibitor, blocked A20 expression under hypoxia at the concentration of 10 μmol/l (Fig. 6B). These findings suggest that A20 expression is induced by hypoxia through the NF‐κB activity.

The influence of A20 on NF‐κB activation was determined by NF‐κB/RelA translocation and IκB‐α degradation. In CTRL siRNA cells, hypoxia caused an increase in NF‐κB/RelA in nucleus and a decrease in IκB‐α protein level, which was consistent with the notion that NF‐κB was hypoxia‐responsive. While under hypoxic conditions, A20 siRNA lead to more prominent NF‐κB/RelA in the nucleus and more weakened IκB‐α expression (Fig. 6C and D). In Figure S1C, we have confirmed decreased IκB‐α at the 9th day of hypoxia in the rat lung tissues. Therefore, the effect of A20 on EC regulation and NF‐κB signalling *in vitro* is correlated back to reduced IκB‐α *in vivo*.

In Figure S4A, we confirmed increased hypoxia‐inducible factor (HIF)‐1α protein level under hypoxic conditions. Dimethyloxalylglycine (DMOG) is a stabilizer of HIF‐1α, which leads to the stabilization of HIF‐1α and angiogenesis through inhibiting HIF prolylhydroxylase 33, 34. In addition, DMOG has been demonstrated to regulate the NF‐κB pathway similar to hypoxia, and there is also further evidence that oxygen‐sensing hydroxylases regulate NF‐κB activity. To explore the mechanisms of how hypoxia regulates NF‐κB and A20 in PAECs, we used DMOG (500 μM) instead of hypoxia. It was noted that treatment with DMOG led a similar up‐regulation of A20 as observed with hypoxia (Fig. S4B). Therefore, HIF might play a critical role during hypoxia‐induced NF‐κB signalling and A20 expression.

Based on these data, we confirm that NF‐κB signalling is essential for hypoxia‐induced A20 in PAECs, and endogenous A20 is a suppressor to NF‐κB. This function is considered as a negative feedback loop.

## Discussion

### Key findings in this study

**Figure 7:**
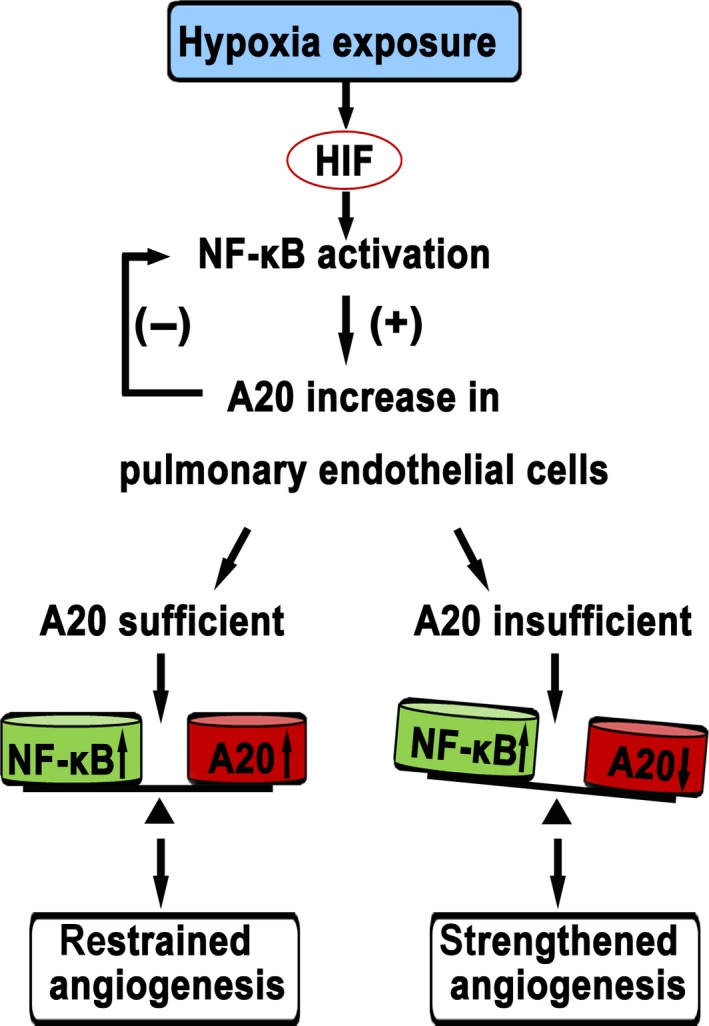
Proposed mechanism of A20 in hypoxia‐induced angiogenesis of PAECs.

The experimental evidence from this study clearly demonstrates that, the elevation and loss of A20 in pulmonary artery endothelium triggered by hypoxia is time dependent. A signalling pathway of the negative feedback loop between A20 and NF‐κB under hypoxia is identified as summarized in Figure 7. During early hypoxia (6–12 hrs), elevated A20 represses the activity of NF‐κB and restrains the angiogenesis of PAECs at least in part. With sustained hypoxia (24–48 hrs), the lack of A20 leads to strengthened NF‐κB activation, pronounced proliferation and angiogenesis.

### The action of A20 in pulmonary endothelium depends on hypoxia

It was previously reported that A20 could be induced by TNF‐α, estradiol, interleukin‐1, CD40 cross‐linking in various cells 11, 18, 35. Emerging evidence indicates that the gene encoding A20 might be associated with hypoxia in glioblastoma cell‐lines, human monocyte‐derived macrophages and primary mouse hepatocytes 20, 21, 36. However, hypoxia has been confirmed as a novel A20 inducer only in few cell types, and the understanding of the relationship between hypoxia and A20 is limited. For example, in four glioblastoma cell‐lines, A20 expression in LN229 and LNZ308, rather than in LN319 and U87, was induced by hypoxia 6, 21.

Hypoxia is known to induce vascular abnormalities and contribute to the development of PAH 6. Thus, we questioned whether A20 would be affected during hypoxia‐induced PAH. We found a transient augmentation of A20 in vascular endothelium following hypoxia, which declined accompanied by vascular abnormalities. When A20 was induced during the 3rd to the 5th day of hypoxia, there was no significant change in the vasculature. This period was supposed to be an early stage of hypoxia. It is possible that the rapid and early increase of A20 may allow the vasculature to maintain a normal state. In fact, enhanced A20 by cytokines is regarded as an early response in primary islets and insulinoma cells 37. A20 has also been reported to be an early responsive factor of Toll‐like receptor 5 signalling in intestinal epithelial cells 38. Thus, the functional role of A20 in hypoxic PAECs elicited a great interest.

### Essential role of A20 in hypoxia‐induced angiogenesis of PAECs

Numerous investigators have tried to shed light on the impact of A20 in apoptosis and proliferation, although the conclusions are controversial. For example, a cyto‐protective role of A20 was identified upon suppressed apoptosis and ECs activation 12, 32, 35. However, this is in contrast with the observation that A20 ameliorates the neointimal hyperplasia after balloon injury *via* protecting against proliferation and migration of vascular SMCs 19, 39. Similar results show that A20 deficiency reinforces the pro‐survival signals and promotes the anti‐apoptotic proteins in cultured dendritic cells 13. These information suggest that the effects of A20 may be different and even opposite depending on the cell types and stimuli.

Disordered proliferation of PAECs under hypoxia results in angiogenesis and proliferative plexiform lesions, which are crucial pathological changes in PAH 5, 6. Therefore, it was proposed that the induction of A20 under hypoxia could potentially play an important role during the progression of this pathology. Here, we demonstrate that A20 silencing leads to increased PAEC cells proliferation, enhanced cell transition from G_0_/G_1_ phase to S phase, increased cell cycle proteins, and reduced cyclin‐dependent kinase inhibitors by A20 silencing. Previous reports indicate that in breast cancer cells and SMCs, A20 regulates cell cycle‐related molecules and these findings are in agreement with our results 18, 39.

A20 is known to restrain the migration of SMCs 19. Besides, tube formation in primary human umbilical vein ECs is proved to be suppressed following A20 inhibition 40. Here, we found A20 silencing led to more enhanced migratory capability and tube length. In the proliferation tests, it was demonstrated that sufficient A20 during hypoxia was quite effective to restrict cell growth in a normal state. But, there was a significant increase in cell migration and tube formation in the hypoxic group with abundant A20, compared to the normoxic control. Taken together these results suggest that A20 exerts a protective effect which is not enough powerful to completely block migration and tube formation of PAECs under hypoxia. We also found that A20 siRNA induced exaggerative ICAM‐1 and VCAM‐1 expression in PAECs. This is consistent with the previous report that cell adhesion molecules are implicated with A20 in aortic ECs and SMCs 39, 41.

Our data demonstrate that the exaggerative expression of A20 plays a considerable role in restricting hypoxia‐induced proliferation and angiogenesis of PAECs, at least in part. This effect is transient as A20 declines quite soon. Therefore, A20 is regarded as an early adaptive factor during hypoxia. A20 has also been confirmed to be an early responding negative regulator of Toll‐like receptor 5 signalling in intestinal epithelial cells 38.

### The reciprocal regulation of A20 and NF‐κB under hypoxia

In Jurkat T cells and β‐cells, researchers have documented the contribution of the transcriptional factor NF‐κB to activate A20 23, 37. It has been reported that there is a negative regulation of NF‐κB signalling by A20 in aortic ECs 32. Recently, it has been proved that in cultured PAECs, NF‐κB activity is rapidly evoked by the treatment of hypoxia 22, 42. It seems likely that increased A20 in PAECs during hypoxia is associated with the activation of NF‐κB.

Through administration of a transcriptional inhibitor, we found that the expression of A20 was transcription‐dependent, which suggests that there might be some transcriptional factors responsible for hypoxia‐induced A20. And NF‐κB was proved to be necessary for A20 expression. As A20 was able to inhibit NF‐κB activity, we conclude that there is a reciprocal regulation of NF‐κB and A20 in PAECs. Thus, it is possible that loss of A20 during sustained hypoxia might result in prolonged NF‐κB activity.

Nuclear factor‐kappa B is traditionally known as a NF implicated in inflammation. Recently, researchers have revealed a novel role for NF‐κB in promoting angiogenesis of primary pulmonary ECs. The mechanism underlying is a direct regulation of VEGF‐receptor‐2 by NF‐κB 43. The inhibition of A20 against NF‐κB translocation and activation was suggested to be depending on a level upstream of IκB‐α degradation 41, 44. Accordantly, we demonstrated that A20 blocked NF‐κB and angiogenisis by regulating NF‐κB/RelA translocation and IκB‐α degradation. It was speculated that the rapid and transient enhancement of A20 might maintain a balance with the function of NF‐κB, restrain the development angiogenic events at least in part, and act as an early adaptative factor during hypoxia.

HIF is the main transcription factor activated by hypoxia and plays an important role in hypoxia‐induced angiogenesis. It has been reported that overexpression of HIF increases proliferation, migration and tube formation of human PAECs, which contributes to hypoxic pulmonary hypertension and vascular remodelling 45. In the present investigation, we found that A20 impairment led to enhanced angiogenic events, and HIF‐1α might play a pivotal role during hypoxia‐induced NF‐κB signalling and A20 expression.

## Conclusions

The present findings demonstrate that in pulmonary artery endothelium, repressed A20 leads to abnormal angiogenesis, and elevated A20 is a significant contributor to restrict proliferation and angiogenesis at least in part during the early stage of hypoxia. The action of A20 is mediated by a close interplay with NF‐κB. On the basis of the critical role of A20 in pulmonary vasculature, we speculate that during the pathogenic process of PAH, additional increase in A20 might alleviate angiogenesis and vascular remodelling, although further evidence for *in vivo* verification is still needed. Thus, a novel option focusing on A20 is provided to design therapeutic strategies for PAH in the future.

## Conflicts of interest

There are no conflicts of interest.

## Supporting information


**Figure S1** The development of pulmonary vascular changes from hypoxic (0, 3, 5, 9 days) rats is time dependent.
**Figure S2** The effect of siRNA against A20 on cell viability during sustained hypoxia (24 hrs).
**Figure S3** Overexpression of A20 restrains cell viability under hypoxia.
**Figure S4** Role of HIF‐1α during hypoxia‐induced A20 expression.
**Data S1** Materials and methods.
