# Supplementary material for: A20 deficiency leads to angiogenesis of pulmonary artery endothelial cells through stronger NF‐κB activation under hypoxia
Source: J Cell Mol Med. 2016 Mar 17;20(7):1319–28. doi: 10.1111/jcmm.12816 (PMC4929300; doi:10.1111/jcmm.12816)
Supplement: Supplementary file 1 — Figure S1 The development of pulmonary vascular changes from hypoxic (0, 3, 5, 9 days) rats is time dependent. Figure S2 The effect of siRNA against A20 on cell viability during sustained hypoxia (24 hrs). Figure S3 Overexpression of A20 restrains cell viability under hypoxia. Figure S4 Role of HIF‐1α during hypoxia‐induced A20 expression. Data S1 Materials and methods. [file JCMM-20-1319-s001.pdf]

ONLINE SUPPLEMENT

A20 deficiency leads to angiogenesis of pulmonary artery endothelial cells through stronger NF-κB activation under hypoxia

Jing Li[1,2], Linlin Zhang[1], Yueming Zhang[1,2], Ying Liu[1,2], Hongyue Zhang[1,2], Liuping Wei[1,2], Tingting Shen[1,2], Chun Jiang[3] and Daling Zhu[1,2]*

[1]*Department of Biopharmaceutical Sciences, Harbin Medical University -Daqing, Daqing, Heilongjiang, China*

[2]*Department of Biopharmaceutical Key Laboratory of Heilongjiang Province, Harbin Medical University, Harbin, Heilongjiang, China*

[3]*Department of Biology, Georgia State University, Atlanta, Georgia, United States*

***Correspondence to:**

Dr. Daling Zhu

College of Pharmacy, Harbin Medical University (Daqing)

Xinyang Road Gaoxin District

Daqing, Heilongjiang 163319 P. R. of China

Tel: +86-459-8153555

Fax: +86-459-8153556

E-mail: dalingz@yahoo.com

Materials and Methods

Materials

Specific antibodies for A20, PECAM, α-SMA, cyclinE and VCAM-1 were purchased from Santa Cruz Biotechnology Inc. (Santa Cruz, USA). GAPDH, p21, p27, IkBα, NF-κB/RelA and hypoxia-inducible factor (HIF)-1α antibodies, as well as actinomycin D (ActD) and BAY11-7082 were obtained from Beyotime Institute of Biotechnology (Haimen, China). Anti-proliferating cell nuclear antigen (PCNA), anti-α-tubulin, anti-cyclinD, and anti-ICAM-1 were from Boster Biological Technology Co. Ltd (Wuhan, China). DMOG was purchased from Cayman Chemical Company (Ann Arbor MI, USA). Lipofectamine 2000 was from Invitrogen (New York, USA). BrdU proliferation assay kit was purchased from Millipore Corporation (Billerica, MA). The CycleTEST™ PLUS DNA Reagent Kit and growth factor-reduced Matrigel were obtained from BD Biosciences (Bedford, MA). All other reagents were from common commercial sources.

Western Blot

Proteins from tissues and cells were extracted with cold lysis buffer (Tris 50 mM, pH 7.4, NaCl 150 mM, Triton X-100 1%, EDTA 1 mM, and PMSF 2 mM) according to the protocol we published before [25]. Following ultrasonication and centrifugation, the supernatant was harvested. Protein concentration was determined using the Bio-Rad protein assay kit (Bio-Rad Laboratories, Inc., Berkeley, CA, USA). Subsequently, 20 µg protein samples were electrophoresed on 8%-10% SDS-PAGE, and transferred onto nitrocellulose membrane (Millipore, USA). After blocked with 5% nonfat milk in Tris-buffered saline buffer (20 mM Tris, 150 mM NaCl, pH 7.6 Tween20 0.1%), the membranes were incubated with primary antibodies at 4°C

overnight, and reacted with horseradish peroxidase-conjugated secondary antibodies. Blot bands were developed by enhanced chemiluminescence reagents (Amersham, UK).

Lung Histology

Lung tissues were fixed with 4% paraformaldehyde, embedded in paraffin and sectioned. Tissue sections were stained with hematoxylin-eosin to determine the severity of pulmonary vascular remodeling. After antigen plerosised and endogenous peroxidase activity blockade, sections were incubated with specific antibody against A20 at 4°C overnight, reacted with a rabbit secondary antibody at room temperature for 20min, visualized using 3,3-diaminobenzidine and counterstained by hematoxylin. Brown and yellow colors represented positive stainings. For frozen slices, fixed tissues were embedded in OCT, double-stained with A20 and PECAM antibodies, incubated with Cy3 or FITC coupled secondary antibodies, and counterstained by DAPI as we described recently [26]. The stainings were examined under a fluorescence microscope (Nikon).

RT-PCR

Total RNA was extracted from prepared PAECs with Trizol reagent (Invitrogen), and reverse-transcribed using Superscript first-strand cDNA synthesis kit (Invitrogen).The cDNAs were amplified with primers according to the procedure we reported before [26]. Designed gene-specific primers were as follows: A20/*TNFAIP3* (GenBank accession no. NM_001192170.1):sense: 5'-GGATAGCCTCGTCAAGATG-3',antisense: 5'-CCACTGTCCTTCATTGTCA-3', length: 297 bp. β-actin (GenBank accession no.NM_173979.3)：sense:

5’-TCCGTGACATCAAGGAGAAGC-3’, antisense:5’-GCACCGTGTTGGCGTAGAG-3’, length: 260 bp. The PCR products were detected by ethidium bromide-stained agarose gel electrophoresis. Images were obtained with agel imaging analysis system (Alpha Innotech, San Leandro, CA). β-actin was used as an internal control.

Immunocytochemistry

Prepared PAECs were fixed with 4% paraformaldehyde for 15 min, permeabilized with 0.5% Triton X-100 for 10 min, and blocked with 5% normal bovine serum for 30 min. After that, samples were incubated with anti-A20, anti-NF-κB/RelA or anti-α-tubulin primary antibody at 4°C overnight. Then secondary antibody (Cy3 or FITC) was incubated for 1 hour in dark, and DAPI was counterstained for 5 min. Samples were examined with a fluorescence microscope (Nikon).

siRNA and plasmid Transfection

To silence A20 expression, PAECs were transfected with siRNA using X-treme Gene siRNA Transfection Reagent (Roche Applied Science, Mannheim, Germany). The siRNA was designed and synthesized by GenePharma. The sequences of siRNA against A20, and non-targeted control were: A20 siRNA (NM_001192170.1): 5’-GCAUCUGCAGUACUUGCUUTT-3’, and CTRL siRNA (non-targeted control): 5’-UUCUCCGAACGUGUCACGUTT-3’. A20 over-expression was performed by transfecting pCMV-A20 plasmid with lipofectamine 2000. The control and pCMV-A20 plasmid was synthesized by Genechem (Shanghai, China). In serum-free Opti-MEM-1 medium,2 µg siRNA was incubated with X-tremeGene, and 1 µg plasmid was incubated with lipofectamine 2000for 20 min according to

manufacturer’s instructions. The mixture was added directly to PAECs. After 6-8 hours, the medium was changed to DMEM containing 5% FBS.

MTT Assay

To evaluate the survival rate of cells, PAECs were seeded into 96-well microtitration plates at about 1×10^4 per well. Following a serum starvation for 24 hours, the cells were subjected to different treatments. The aim of a growth arrest is to unify the cells into the same status of growth before treatments, and the cell viability would not be affected. After that, cells were incubated with MTT at a concentration of 0.5% in medium, at 37°C for 4 hours. At the end of this step, the reaction was terminated by removing the supernatant, adding dimethyl sulfoxide, and shaking for 10 min at room temperature. The absorbance was measured at 490 nm in a spectrophotometer.

BrdU incorporation

PAECs were seeded in 96-well plates at a concentration of 1×10^4 cells/well. After starvation for 24 hours, cells were labeled with 10 μM BrdU, exposed to normoxia or hypoxia, and fixed with fixing solution at the end of exposure. Following washing step, anti-BrdU monoclonal antibody was incubated for 1 hour, and goat anti-mouse IgG was conjugated. Thereafter, peroxidase substrate was added, and the reaction was stopped by stop solution. The absorbance of the sample was detected using a spectrophotometer microplate reader at at 450/550 nm.

Cell cycle analysis

The cell cycle progression was measured using the Cycle TEST PLUS DNA Reagent Kit as we reported previously [25]. In brief, prepared PAECs in groups were

trypsinized, harvested and fixed in 70% cold ethanol at 4°C. After discarding the ethanol, cells were resuspended in 200 µl PBS, and incubated with 200 µl propidium iodide at 4°C in dark. Finally, the stained cells were filtered and DNA fluorescence was measured by flow cytometry using BD FACS Calibur Flow Cytometer (Bedford, MA).

Migration Assays

For 24-well Boyden chamber migration assay, DMEM with 10%FBS was in the lower layer of the transwell. Resuspended PAECs in DMEM without FBS were added into the upper chamber at 8×10^4/well. After normoxia or hypoxia exposure, the cells in the 8-µm-pore polycarbonate filter were fixed with 4% paraformaldehyde, and stained with 0.4% crystal violet. Cells in the upper of the filter were removed, and the number of stained migrated cells was counted under an inverted microscope (Nikon).

Tube formation assay

Growth factor-reduced Matrigel (BD Biosciences) was coated in 96-well plates using 30 µl/well and solidified for 30 min at 37°C. Prepared PAECs were trypsinized, resuspended and added onto the Matrigel well at 1×10^4 cells per well. Then the cells were treated with normoxia or hypoxia. Tube formation was examined under an inverted microscope (Nikon). Tube length was measured by Image-Pro Plus 6.0.

Supplemental Figure and Figure Legends

Figure S1

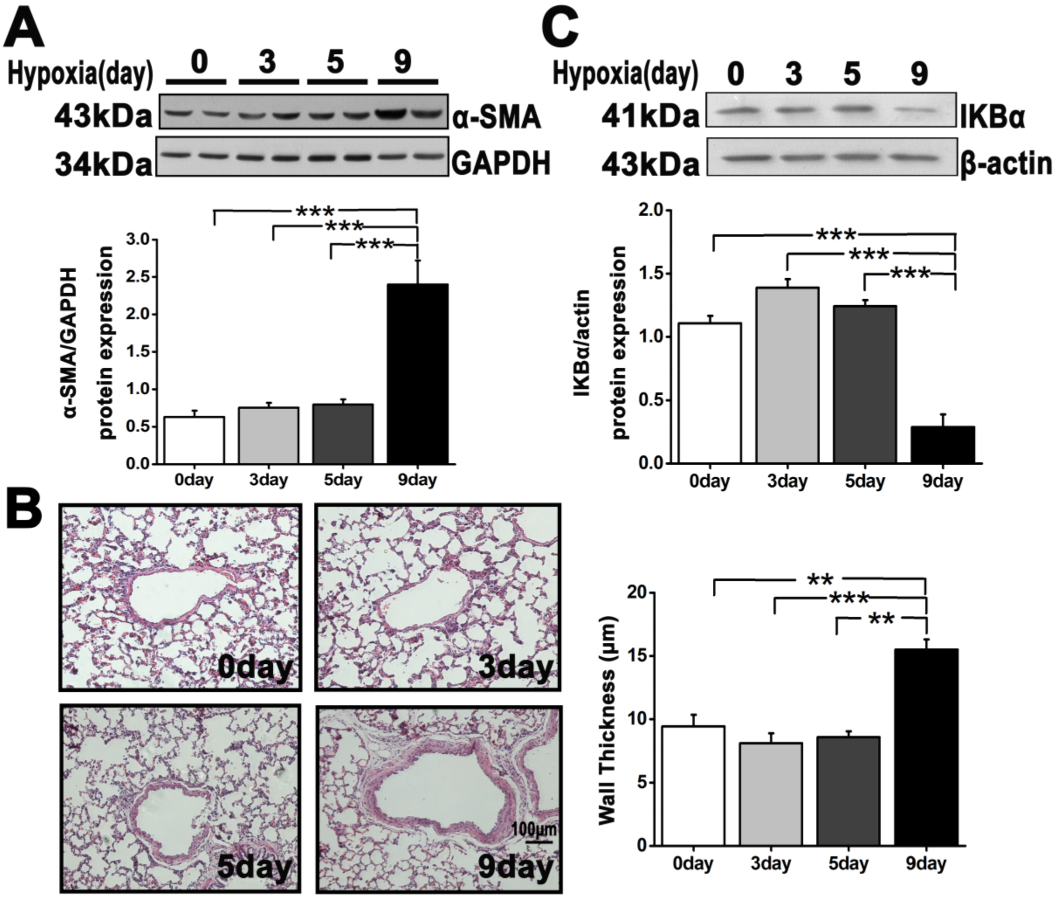

Figure S1. The development of pulmonary vascular changes from hypoxic (0, 3, 5, 9 days) rats is time-dependent . **A)** Western blot analysis of α-SMA protein levels in rat lung tissues (n = 4, ***P < 0.001). **B)** The vascular wall thickness of pulmonary artery was determined by hematoxylin-eosin staining (n = 3, **P < 0.01, ***P < 0.001). Scale bars are 100 μm. Images shown are representative of at least three independent experiments. **C)** IκB-α expression in the lung tissues decreased at the 9th day of hypoxia (n = 4, ***P < 0.001). All the values are represented as mean±s.e.m.

Figure S2

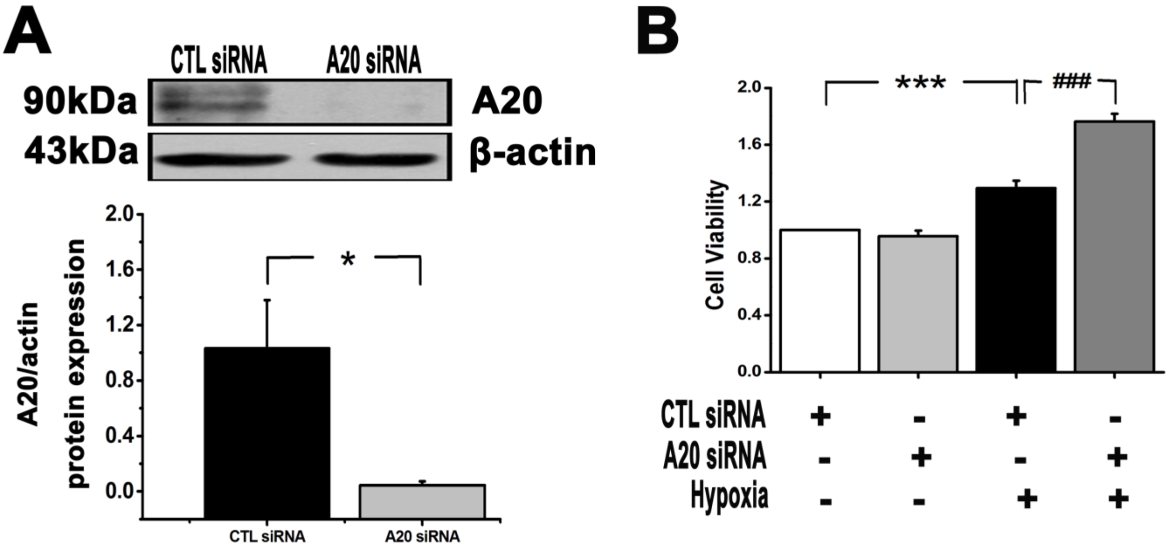

Figure S2. The effect of siRNA against A20 on cell viability during sustained hypoxia (24 hours). **A)** A20 expression was measured after transfection with control or A20 siRNA (n=3, *P < 0.05). **B)** The cell viability of different groups was analysed using the MTT assay (n=7, ***P < 0.001, ###P < 0.001,). "CTL siRNA" means non-targeted control siRNA, and "A20 siRNA" indicates siRNA targeting A20. All the values are represented as mean±s.e.m.

Figure S3

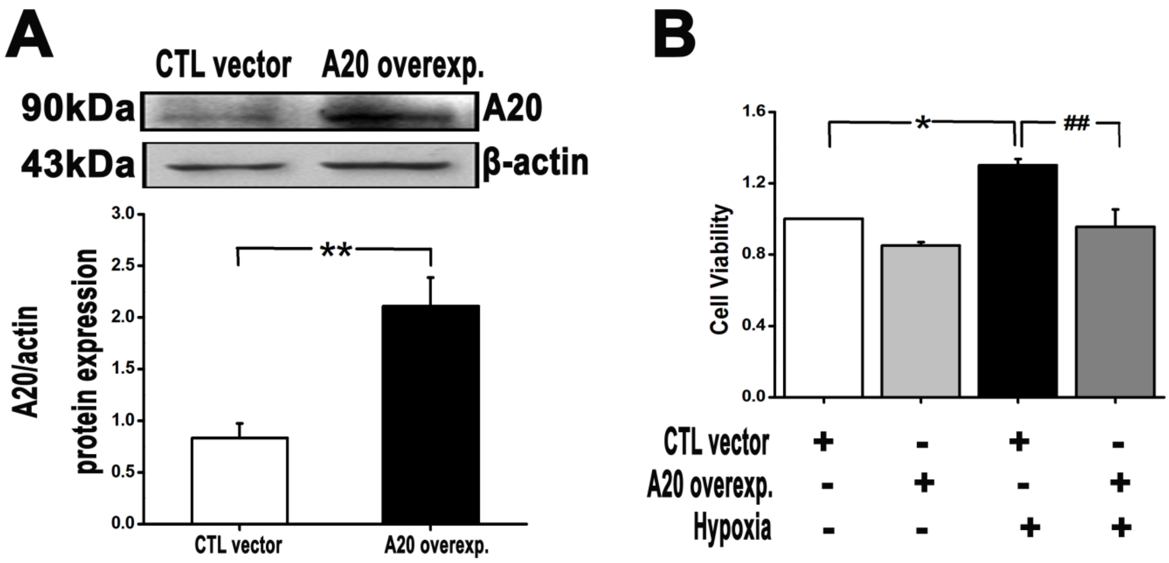

Figure S3. Over-expression of A20 restrains cell viability under hypoxia. **A)** The protein levels of A20 in PAECs transfected with control or pCMV-A20 vector were determined (n=4, **P < 0.01). **B)** MTT assay was performed to detect cell viability after persistent hypoxia (24 hours) (n=11, *P < 0.05, ##P < 0.01). "CTL vector" means control vector, and "A20 overexp." indicates pCMV-A20 expression vector. All of the values are represented as mean±s.e.m.

Figure S4

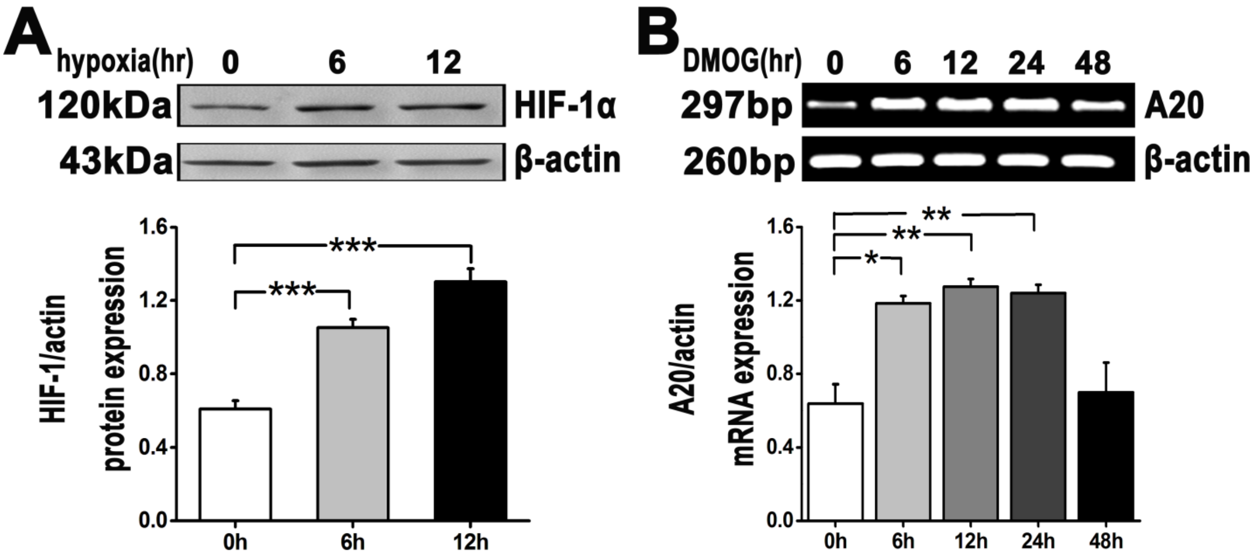

Figure S4. Role of HIF-1α during hypoxia-induced A20 expression. **A)** HIF-1α protein levels in PAECs were measured by western blot analysis (n=4, * **P < 0.001). **B)** PAECs were subjected to DMOG (500μM), and a quantification of A20 mRNA expression at different time points was performed (n=3, *P < 0.05, **P < 0.01). All the values are represented as mean±s.e.m.
